# Peering into the Brain through the Retrosplenial Cortex to Assess Cognitive Function of the Injured Brain

**DOI:** 10.1089/neur.2021.0044

**Published:** 2021-12-02

**Authors:** Helen Motanis, Laila N. Khorasani, Christopher C. Giza, Neil G. Harris

**Affiliations:** ^1^ UCLA Brain Injury Research Center, Department of Neurosurgery, Geffen Medical School, UCLA Mattel Children's Hospital, University of California at Los Angeles, Los Angeles, California, USA.; ^2^ Intellectual Development and Disabilities Research Center, UCLA Mattel Children's Hospital, University of California at Los Angeles, Los Angeles, California, USA.; ^3^ Department of Pediatrics, UCLA Mattel Children's Hospital, University of California at Los Angeles, Los Angeles, California, USA.

**Keywords:** cognitive impairments, hippocampal marker, network activity, neural deficits, sensory-cognitive network, traumatic brain injury

## Abstract

The retrosplenial cortex (RSC) is a posterior cortical area that has been drawing increasing interest in recent years, with a growing number of studies studying its contribution to cognitive and sensory functions. From an anatomical perspective, it has been established that the RSC is extensively and often reciprocally connected with the hippocampus, neocortex, and many midbrain regions. Functionally, the RSC is an important hub of the default-mode network. This endowment, with vast anatomical and functional connections, positions the RSC to play an important role in episodic memory, spatial and contextual learning, sensory-cognitive activities, and multi-modal sensory information processing and integration. Additionally, RSC dysfunction has been reported in cases of cognitive decline, particularly in Alzheimer's disease and stroke. We review the literature to examine whether the RSC can act as a cortical marker of persistent cognitive dysfunction after traumatic brain injury (TBI). Because the RSC is easily accessible at the brain's surface using *in vivo* techniques, we argue that studying RSC network activity post-TBI can shed light into the mechanisms of less-accessible brain regions, such as the hippocampus. There is a fundamental gap in the TBI field about the microscale alterations occurring post-trauma, and by studying the RSC's neuronal activity at the cellular level we will be able to design better therapeutic tools. Understanding how neuronal activity and interactions produce normal and abnormal activity in the injured brain is crucial to understanding cognitive dysfunction. By using this approach, we expect to gain valuable insights to better understand brain disorders like TBI.

## Introduction

The past few decades of research have established that network-level phenomena are commensurate with cognition and behavior^1–3^ and that disruptions of normal network activity across the brain are associated with many neuro- and neuropsychiatric diseases.^4–6^ Traumatic brain injury (TBI) is one such disease that has been associated with significant deficits in network dynamics^7–10^ as well as motor, cognitive, and emotional deficits. However, deciphering and understanding network alterations associated with TBI can be challenging because of many factors, including that TBI is typically diffuse, affecting multiple cortical and subcortical brain circuits and networks. Fully understanding these multiple network alterations and their clinical implications is difficult, and pre-clinical modeling can be challenging because of the invasiveness of procedures necessary to access subcortical regions classically associated with cognition. In this review, we suggest an alternative approach to investigate cognitive dysfunction post-TBI using the retrosplenial cortex (RSC) as a surrogate structural and functional conduit of the hippocampus, one of the main brain regions underlying cognitive deficits after TBI.

We posit that, to improve understanding of the complex cognitive dysfunction that occurs after TBI, we need to take advantage of this easily accessible, superficial brain region as a proxy for deep brain function to monitor cognitive deficits and response to intervention during ongoing behavioral tasks that produce a heavy functional burden on the brain. More specifically, by utilizing this region for *in vivo* analysis of alterations in functional connectivity that can occur during cognitive tasks—for example with electrophysiology, optical, or ultrasound imaging—the pre-clinical TBI field may gain a valuable tool with which to assess cognitive deficits beyond the typical separate analysis of behavior and functional connectivity during resting conditions. To that end, we first discuss the challenges associated with understanding cognitive dysfunction after TBI by reviewing the pertinent literature. We then expound upon both the benefits and limitations of studying the RSC as a cognitive marker of the hippocampus specifically post-TBI.

## Altered Function after Traumatic Brain Injury

TBI impacts >50 million persons worldwide annually,^11^ resulting in >60,000 deaths in 2017 in the United States alone.^12^ The extent of TBI's brain disruption and prognosis are dependent on a diverse array of factors, including: severity of injury and/or manner of impact,^13^ age,^14–16^ sex,^13,17,18^ comorbidities,^13^ and socioeconomic status.^19^ Over the past decades, extensive research has led to the characterization of abnormalities occurring post-TBI, with many reports indicating underlying deficits in cellular function as well as cell death.^20–22,23–25^ These deficits and others are ultimately manifested in the form of abnormal network dynamics that are associated with altered behavioral patterns and cognitive deficits.^26^

Despite decades of research aimed at clinical interventions and rehabilitation programs for TBI, there are still no U.S. Food and Drug Administration–approved therapeutics to restore neural function or even positively alter the trajectory of the disease.^27–30,31,32^ One major reason for these limited successes is attributable to a fundamental gap in our knowledge of the exact mechanisms in which TBI and concussion alter brain function.^33,34^ Understanding how TBI affects neural dynamics and deciphering the underlying mechanisms of abnormal network activity is fundamental for the design of novel therapeutic targets and interventions.^35^ Pre-clinical TBI research has been foundational toward this goal. Studies using murine-TBI models have recapitulated many of the functional alterations post-TBI and shed light into the mechanisms underlying these deficits.^36–42,43^ Among many of the structural deficits reported in animal models of TBI,^44,45^ cell death,^46,47^ white matter damage,^31,39–43,52,36^ and tissue atrophy^53,54^ are among the most evident^55^ to contribute to irreversible damage of brain circuits, as indicated by persistent deficits in network dynamics.^34^ This becomes particularly relevant for the study of how injury affects the ability of the brain to maintain normal network dynamics and to our ability to intervene, stop, or rescue deficits of neural dynamics post-TBI in all severities.^7,56^

## Hippocampal-Dependent Impairments after Traumatic Brain Injury and Gaps in Knowledge

The hippocampus has been identified as one of the most important brain regions that contributes to post-TBI cognitive deficits, positioning it as high interest for TBI research.^57–64^ In particular, the hippocampus has been shown to be susceptible to cell death post-injury,^65^ contributing to significant pathological, structural, and functional alterations,^59,66–71^ along with other post-injury learning and cognitive deficits.^45,72–75^ For example, neuroimaging studies have confirmed hippocampal microstructure alterations,^76,77^ reduced hippocampal-cortical connectivity,^37,78^ and white matter deficits^79^ post-TBI. It is notable that, after injury, neural alterations and hippocampal hyperexcitability^80,81^ have also been shown to contribute to epileptic-like activity.^82,83^

Not surprisingly, TBI and concussion have been shown to cause declines in a set of hippocampal-dependent cognitive tasks both in humans^84–87^ and in TBI animal models.^64,74,75,88^ For example, multiple studies utilizing the Morris water maze (MWM) to understand cognitive deficits post-TBI have shown learning and memory impairments post-injury.^64,74,89^ Similar deficits were reported in other memory tasks, such as the radial arm maze,^90^ Y maze,^91^ and object recognition tasks.^92^ Most interestingly, deficits were also reported when the hippocampus was not at the primary site of injury, indicating that diffuse impacts of TBI can also contribute to a marked loss of function in hippocampal-dependent tasks.^89,93^ This finding has also been reported in TBI-patients,^73,94^ further establishing the hippocampus and, presumably, functionally connected regions as important brain regions altered post-TBI.

Accordingly, the hippocampus serves as a valuable indicator of cognitive decline post-TBI.^61,63^ However, when reviewing the TBI literature, there remains a clear gap in knowledge regarding the cellular-level, hippocampal-dependent behavioral alterations post-TBI and the mechanisms underlying them. A greater understanding of how cells interact to produce network-level activity post-TBI is crucial for parsing the mechanisms of post-trauma neural deficits and how they relate to cognitive dysfunction.

To the best of our knowledge, there have been only a small number of studies examining hippocampal neural activity *in vivo* and in awake animals under task conditions after TBI.^95–101^ For example, theta power and theta peak frequency, two hippocampal correlates of behavior, were attenuated post-TBI and were correlated with poor performance on the Barnes maze.^96^ In addition, hippocampal single-neuron activity was also altered during a memory task even when a lack of morphological change was reported after a mild form of TBI.^95^ Another study reported a significant reduction in place cells number and the spatial features of the remaining place cells after fluid percussion injury,^99^ whereas recovery of theta frequency oscillations occurred commensurate with improved behavior.^101^ Other studies that investigated neural activity *in vitro* or *ex vivo* reported long-term potentiation alterations,^102^ hyperexcitability and reduced threshold for seizure-like activity,^80^ and synaptic deficits,^103^ in agreement with *in vivo* deficits and confirming the presence of abnormal synaptic and network activity post-TBI.

## *In vivo* Recording of Hippocampal Function and State-of-the-Art Alternatives

Despite the great advancement in our understanding of the role and alterations of the hippocampus post-TBI, many questions remain unanswered. For instance, how does hippocampal dysfunction directly affect behavior, and how is it manifested post-TBI? Studying these questions and others ideally *in vivo* and in awake models of TBI can reveal some of these mechanisms, but unfortunately scientists in the field have been facing many challenges, mainly attributable to caveats that make the hippocampus physically less accessible. Although task and resting-state functional (fMRI) magnetic resonance imaging (MRI) are well-established modalities for providing network-level information relating to brain function, they are not specific to cell type, and important questions remain regarding the state of neurovascular coupling after TBI that might render acutely acquired imaging information inaccurate.

Additionally, surface-level recording tools like electroencephalography (EEG) generally only capture superficial brain areas. The hippocampus is positioned deep in the brain, making it physically inaccessible for novel recording tools, such as cellular-level imaging, that require proximity to the tissue being recorded. A notable widespread modification to overcome this issue in the field of two-photon calcium imaging is physical aspiration of the cortical regions above the hippocampus.^104–107^ This allows a direct access to the hippocampus, and many research groups have adopted this method to conduct studies aimed at understanding how the hippocampus performs certain computations. However, this is a very intrusive technique that affects both the anatomy and the function of the brain and adds many confounding factors. Such modifications are required until the field develops better techniques and may be appropriate in studies that are less clinically applied or where the major focus is on specific circuit-level analysis.

Given that TBI can affect the whole brain, a cellular-level analysis requires a less-intrusive technique for the acquired data to truly reflect the multiple number of networks involved in cognitive dysfunction. Herein, we suggest an alternative strategy for investigating hippocampal dysfunction by the study of a surrogate brain area that is positioned on the surface of the cortex, and is both interconnected with the hippocampus and well known to be involved in cognitive function. After a careful examination of the literature, we propose the RSC as a model candidate for such a strategy to serve as a reliable indicator of cognitive dysfunction after TBI.

## The Retrosplenial Cortex Can Act as a Cortical Alternative for the Hippocampus

Here, we review the literature to answer the question of whether studying and understanding neuronal activity in the RSC can serve as a readout of activity in the hippocampus. More specifically, we focus on the RSC as a more accessible alternative to study neuronal dynamics post-TBI.

In rodents, the RSC is located on the surface of the brain in the caudal dorsal region and can be easily accessible using different invasive experimental methods and imaging as reviewed previously.^108^ In addition, newer experimental technologies, such as two-photon microscopy wideband optical imaging and functional ultrasonography, can also be leveraged. Although our understanding of the role of the RSC is still in its infancy, there has been a rapidly increasing interest in this region over the past few years because of the discovery of a large array of functions associated with the RSC. These functions include, but are not limited to, spatial navigation,^108–110^ episodic memory,^108,111^ and multi-modal sensory and path integration.^108,112–115^ These studies provide important clues to the functions of the RSC and suggest that it may be optimally located to report on neural activity related to cognitive deficits post-TBI. We review a number of these articles and provide an overall perspective on how the RSC can serve as a cognitive marker of the hippocampus specifically post-TBI.

## The Anatomical Connections of the Retrosplenial Cortex: A Major Cortical-Hippocampal Conduit

The global anatomical connections of the brain are most often represented as a structural connectome. After TBI, the connectome is altered because of a combination of focal and diffuse damage to axonal projections.^116^ The RSC is a critical source and recipient of information to and from a broad array of brain regions with altered connectivity after TBI. To this end, it is important to appreciate the anatomical location and connectivity of the RSC in the naïve brain. In rodents, the RSC is characterized as a higher-order association area that can be further divided into two subregions based on cytoarchitecture and connectivity; granular (RSCg) and dysgranular RSC (RSCdys),^108,117^ or described by anatomical terms such as dorsal and ventral RSC (RSCv), respectively. In some literature, the RSCdys has been referred to as agranular RSC (RSCagl)^118^; however, for the purposes of this article, we primarily rely on the same naming conventions described previously^108^ along with the anatomical terminology, when relevant.

**FIG. 1.**
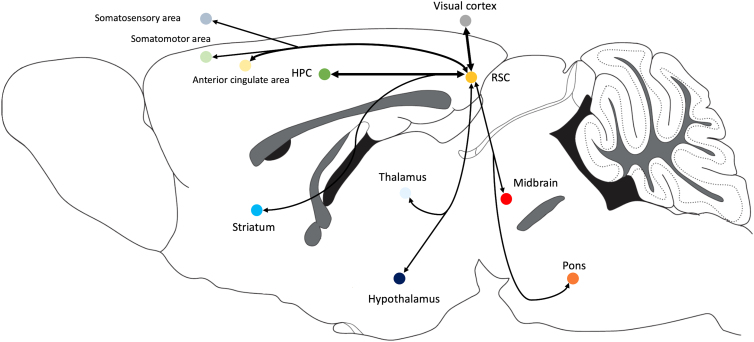
Schematic depiction of the major anatomical pathways that connect the RSC to the brain displayed on a mid-sagittal section of the rat brain. Colored nodes indicate brain regions with anatomical connectivity with the RSC. Areas that display high levels of connectivity are displayed by thicker arrows. HPC, hippocampus; RSC, retrosplenial cortex.

Studies using anatomical tracing and neuroimaging in rodents have shown that the RSC has extensive and diverse connections with many brain regions (Fig. 1), including; parietal association cortex,^119,120^ somatosensory and parietal cortex,^121,122^ visual cortex,^120,121,123^ auditory cortex,^121^ broader neocortex,^124^ motor cortex,^125,126^ anterior cingulate cortex,^121,123,124,127^ hippocampus,^108,119,120,124,128,129^ entorhinal cortex,^121,123,125,127^ midbrain,^119,120,124,127^ thalamus (anterior and laterodorsal nucleus),^108,119,120,123,124,127,128,130^ hypothalamus,^119,120,123,124^ and some smaller connectivity with the striatum^123,124^ and pons.^123^ Overall, the diversity and complexity of these connectivity patterns suggest that the RSC may be optimally located within the brain to integrate sensory and cognitive information, establishing its role as an essential circuit in the sensory-cognitive network.^108,109,118,119,123,131,132^ Below, we summarize the anatomical evidence supporting this hypothesis, and in the following sections, we elaborate on the functional and behavioral studies that establish the RSC as a sensory-cognitive integrator.

**FIG. 2.**
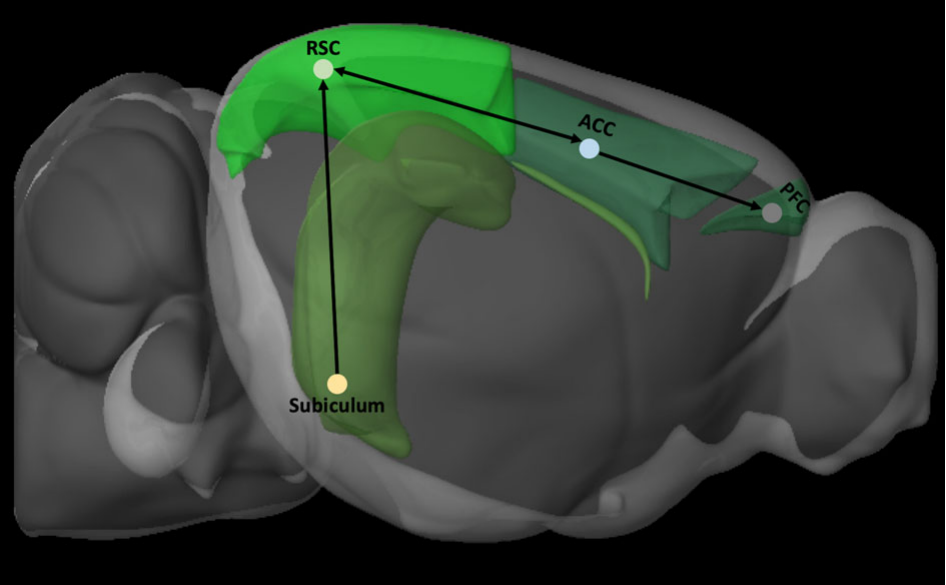
Schematic depiction of the trisynaptic circuit by which information from the subiculum is transmitted to the cortex by the RSC. Brain regions were defined by Allen Brain Explorer 2 software (Allen Institute for Brain Science, Seattle, WA). ACC, anterior cingulate cortex; PFC, pre-frontal cortex; RSC, retrosplenial cortex.

One major route through which information processed within the dorsal hippocampus can reach the neocortex is through the RSCv, which is the only neocortical recipient of dense inputs from the dorsal subiculum.^133^ From here, the RSCv can project this information to the medial pre-frontal cortical areas,^121,134–137^ through massive reciprocal connections between the RSCv and the ventral anterior cingulate area^137^ (Fig. 2). The entorhinal cortex is also a major point in this trisynaptic circuit; before information from the RSC reaches the dorsal subiculum.^138^ This juxtaposition establishes the RSC as part of a trisynaptic circuit (Fig. 2) that connects the hippocampal region to the neocortex^125,139^ and forms the basis for the RSC serving as a critical anatomical conduit through which potential information regarding cognition, navigation, episodic memory, and other hippocampal-dependent functions reaches the neocortex.^108,133,140^ This positions the RSC at an optimal location for understanding cognitive dysfunction after TBI because of the high number of incidents of injuries affecting one or multiple brain regions in this trisynaptic circuit.^141,142^

The effects of TBI can also be investigated by studying alterations occurring directly within the RSC. The documented alterations in RSC connectivity that occur post-TBI,^143^ predominance of hippocampal-related behavioral deficits, and central position of the RSC within these circuits support the idea that the RSC contributes significantly to hippocampus-dependent functions. As such, studying the RSC would therefore provide a useful and easily accessible platform to study circuits related to cognitive dysfunction.

**FIG. 3.**
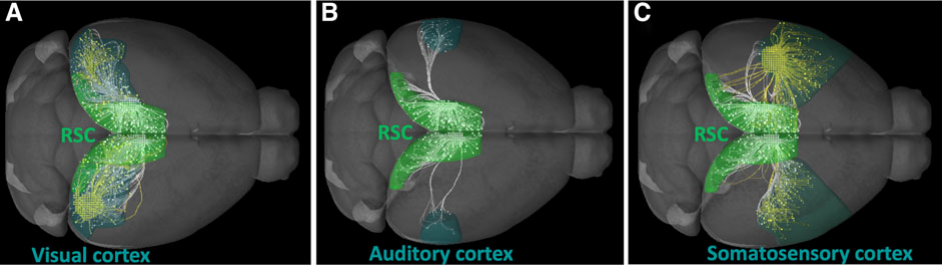
Representative images of RSC anatomical projections to and from various cortical areas. Projections were acquired from data in the Allen Mouse Connectivity experimental database, of tracer experiments using rAAV tracers and two-photon topography, and shown using Allen Brain Explorer 2 software (Allen Institute for Brain Science, Seattle, WA). (**A**) Representative tracer experiment showing projections between the RSC and visual cortex. (**B**) Same as in (A), but projections are between the RSC and auditory cortex. (**C**) Same as in (A), but projections are between the RSC cortex and somatosensory cortex. Yellow projections originate in each respective region and travel toward the RSC. White projections originate in the RSC and travel to the designated region. rAAV, recombinant adeno-associated virus; RSC, retrosplenial cortex.

Outside of the hippocampal formation, the RSC also holds strong reciprocal anatomical connectivity with cortical areas (Fig. 3), such as the primary visual cortex (Fig. 3A).^121,124,144–146^ The RSC-visual cortex reciprocal circuit has been receiving increasing attention in recent studies demonstrating the importance of the RSC as an integrator of sensory information to predict and inform on behavioral outcome.^147,148^ This is particularly important in TBI where sensorimotor and visual deficits are part of the TBI sequela.^149^ Accumulating data have indicated that visual deficits that are not associated with structural damage to the eye may emerge after TBI,^150,151^ including blurry vision,^152,153^ convergence insufficiency (inability of two eyes to work together),^154,155^ and visual field loss.^156^ Whereas these deficits are largely attributable to alterations in the acquisition of visual information, there is also evidence suggesting that TBI induces delays in visual processing in humans,^157^ likely implicating central circuits that may involve the RSC.

Other cortical areas are also interconnected with the RSC. For example, the RSCdys and RSCagl receive sparse projections from the auditory (Fig. 3B) and somatosensory (Fig. 3C) cortices.^121^ This further implicates the RSC as a relay for sensory information and may underly the well-known motor, balance, and deficits in coordination after TBI.^158–160^ In addition, it has also been shown that the RSC is connected to the primary and secondary motor cortices^134^ and that it also receives projections from the motor areas in a topographical manner.^135^ The presence of the RSC/motor cortex circuit suggests that the RSC may play a role in motor-related behaviors.

## Functional Connectivity of the Retrosplenial Cortex

Thus far, past anatomical information indicates that the RSC is highly connected with many brain regions. Further, the RSC has also been identified as an important node in the default-mode network (DMN),^161–163^ a network of functionally connected and coordinated brain regions that have been used to describe the brain's “intrinsic” activity patterns.^164^ Alterations to either cortical or subcortical nodes of the DMN can alter the broader functionality of the entire network,^165^ and abnormalities in the DMN have been associated with numerous brain disorders such as schizophrenia^166^ and Alzheimer's disease (AD).^167^ The medial temporal lobe (MTL) and the RSC, two nodes in the DMN, have been invoked in humans to explain why AD patients show reduced metabolism in the RSC during early stages of the disease when the pathology is limited to the MTL.^167^

Additionally, increased connectivity between the RSC and pre-frontal cortex (PFC), another important node in the DMN, has been associated with sleep disturbances.^168^ Importantly, diffuse axonal injury and white matter damage post-TBI have been shown to directly affect the functional connectivity between different nodes of the DMN.^169,170^ These studies indicate that TBI alterations can significantly affect dynamics of the DMN, and because both the hippocampus and RSC are important nodes in this network, studying either of these brain areas post-TBI is of prime importance to assess the neural network basis of dysfunction and recovery.

## Functions of the Retrosplenial Cortex

The human RSC has been implicated in a variety of brain functions, with a particular emphasis on navigation and memory.^108,112–114,171–174^ As described previously, the RSC is anatomically highly connected to the hippocampus^139^ (Fig. 1), contributing to its involvement in hippocampal-associated functions. Lesion studies or damage to the RSC, both in human patients and animal models, have been shown to produce symptoms of retro- and anterograde amnesia.^108,175–178^ In addition, the human RSC has been shown to be important for episodic-like memory in which elemental information about the what, where, and when aspects integrate^173,179,180^ and also for utilizing environmental cues during navigation.^171–173^ These studies suggest a crucial role of the RSC to recognize integrated entities in terms of their identity,^181–183^ location,^184^ and into the integration of temporal aspects.^185^

Further, the RSC's reciprocal connections with the visual and motor processing areas ideally position the RSC as a brain region that can incorporate visual and motor information for the purpose of path integration. Indeed, Mao and colleagues have shown that visual and locomotion information are incorporated together in the RSC; however, optical information overrides locomotion information.^132^ Not surprisingly, the RSC has also been shown to play a unique role in non-hippocampal functions.^186,187^ Below, we further examine the diverse array of functions associated with the RSC.

## Cognitive Hippocampal-Associated Function

The contribution of the RSC to functions typically associated with the hippocampus first became evident from clinical observations of patients experiencing amnesia post-RSC damage.^179^ This has been further corroborated from observations in AD and cognitive-decline patients.^188^ In 2003, Nestor and colleagues have shown that the RSC exhibits reduced metabolism in patients with cognitive decline and early AD.^189^ In fact, pathological changes in the RSC may be some of the earliest identifiable neurological markers of AD. Atrophy in the RSC has also been observed in early AD.^190^ Of importance is that RSC atrophy is not observed as markedly in behavioral variant frontotemporal dementia as it is in AD,^191^ implying that the RSC may be uniquely involved in AD pathology.

In addition, studies implicating the RSC in AD have been recapitulated in animal models. For example, functional circuit connectivity of the RSC was significantly reduced in rats with cognitive impairments.^192^ Reductions in the MRI signal relaxation parameter, T2, a potential indicator of altered cellular function, were also found in a mouse model of AD within RSC regions in conjunction with hippocampal and cingulate cortex regions.^193^ Last, a more recent study identified the RSC as the region most impacted by amyloid-beta (Aβ) aggregation in an early-AD animal model.^194^

The role of RSC in cognitive decline has also been observed after stroke. Clinically, it has been reported that stroke affecting the RSC is consistent with antero- and retrograde amnesia along with deficits in visual memory.^176^ In experimental stroke localized in the PFC, deficits in spatial memory were observed,^195^ possibly attributable to loss of connections between the PFC, hippocampus, and RSC. Indeed, in an electroacupuncture stroke model, the RSC experienced altered functional connectivity with the hippocampus.^120^ These studies suggest that the RSC will also indicate cognitive deficits post-TBI given that memory and cognitive deficits are among the sequelae of TBI.^74^ Not surprisingly, the RSC has been identified as a region susceptible to cell apoptosis in experimental pediatric TBI models,^196^ and in a study using a mouse-TBI model targeting the sensory cortex, alterations of cerebral blood flow to the RSC were reported post-injury.^197^ In a similar manner to results from AD studies, increased Aβ deposits in the RSC were found after repeated mild TBI, which was exacerbated when consecutive injuries were closer in time.^198^ Although these data indicate that alterations in RSC structure and function occur because of TBI, there remains a need to further investigate RSC pathology after TBI.

Although strong connectivity between the RSC and amygdala has only been corroborated in the macaque,^199,200^ the human RSC was significantly activated when presented with emotionally salient cues,^201^ suggesting that this activation may be attributable to re-encoding of memories associated with the presented cue. This further reinforces the RSC as an important region for stimuli integration and episodic memory. In the human brain, the RSC is also an important integrating center between the MTL and DMN in subjects with strong episodic memory performance.^162^ During autobiographical memory retrieval sessions, the RSC displays strong phase-locking with the MTL during theta frequencies,^202^ and these oscillations have also been observed in the RSC during REM (rapid eye movement) sleep.^203^ Interestingly, some of the strongest evidence of the involvement of the RSC in memory formation comes from RSC lesion studies in rodents, which resulted in alterations in episodic memory retrieval of a delayed-to-matching task and of a tone discrimination task,^204^ whereas chemogenetic inactivation of RSC neurons affected the ability to link sensory stimuli to form episodic memories.^205^

In addition, pharmacological inhibition of N-methyl-D-aspartate receptors in the RSC resulted in altered retrieval of contextual fear memory.^206^ Crucially, for the hypothesis that the RSC can serve as a superficially located conduit for studying memory performance after TBI, episodic memory dysfunction is also a widespread observation in TBI patients.^207–209^ Even in mild cases of TBI, deficits in episodic memory linger and persist, whereas semantic memory and other memory forms recover or are preserved.^210^

## Spatial Navigation and Functional Parcellation of the Retrosplenial Cortex

The TBI literature is replete with publications that describe the learning and memory deficits in spatial navigation using the MWM or Barnes maze. Although this has generally been seen as a hippocampal-dependent process in the TBI field, there is, in fact, a good deal of evidence suggesting that the RSC is involved in these deficits. In fact, the vast majority of RSC studies have focused on studying its role in navigation and have shown that lesions or temporary inactivation of the RSC result in deficits in path integration,^113,114,211^ spatial memory,^108,110,212,213^ and navigating based on only self-motion cues.^211^ Additionally, there is also significant evidence implicating the RSC in cognition and scene construction,^108^ specifically in mediating between different spatial reference frames.^214^ fMRI during virtual-reality navigational tasks indicates a relationship between the hippocampus and RSC when creating and referring to cognitive space maps.^215^

When examining the RSC literature, it is especially important to pay attention to the extent to which the RSC was lesioned or inactivated because it is becoming increasingly apparent that the RSCg and RSCdys play different roles—especially in the context of memory and navigation. For example, the RSCdys is more important for visually guided spatial memory and navigation,^216–218^ whereas the RSCg has been shown to have a greater involvement in internally directed navigation.^216^ This perfectly aligns with the anatomy of the RSC, given that the RSCdys receives large projections from the visual cortex and from subicular areas for navigation-related functionality, whereas the RSCg comparatively receives more auditory,^219^ thalamic,^108^ and postsubiculum.^108^ This also aligns with a study showing that the RSCg mediates spatial navigation in light and dark whereas the RSCdys was involved only in light conditions.^216^ However, further studies are required to fully understand roles of the subdivisions of the RSC because of current conflicting data. For example, a study examining lesions to the caudalmost region of the RSC did not report major deficits in spatial memory,^220^ whereas other studies found that full bilateral lesions of the RSC led to the inability to segregate spatial information and use directional cues appropriately.^221,222^

Given that injury severity or extent of lesion can significantly affect the level of deficit, future TBI research involving the RSC should carefully design experiments that treat location of injury and injury severity as important covariates. Despite these subregions within the RSC that connect disparate information, there appears to be solid support for the causal involvement of the RSC in spatial memory. Evidence from lesion studies also strongly support its use as a superficially located region for monitoring cognitive deficits after TBI.

In relation to its role in navigation, the rat RSC has been also shown to contain head direction cells.^223^ Other studies have shown the existence of head direction cells in the subiculum and entorhinal cortex,^224^ and Sugar and colleagues, in 2011,^139^ suggested that these three brain areas work together to represent head direction in a neural space. This further supports the role of the RSC in navigation, although it remains unclear whether the RSC also has non-spatial contributions. To understand this, a 2004 study investigated the impact of non-spatial pre-training on MWM performance in RSC-lesioned rats. After lesioning, performance of the non-spatial pre-trained animals was significantly better than of those that did not undergo the non-spatial pre-training. These results point to a role of the RSC in cognition when required to respond to a given task appropriately.^225^ Other studies have shown that RSC lesion deficits emerge on non-spatial tasks when animals have to rely on previously acquired representations to solve the current problem or switch between different representations of the same event.^186,205,226–228^

The RSC's strong connectivity with the visual cortex also suggests a role of the RSC as the integration point between visual and navigational information.^229^ Other work has also indicated evidence of cells particular to specific combinations of movement, location, and direction.^230^ Cells in the RSC are also tuned to the location of environmental boundaries in context to the location of self in rats.^231^ Given the emphasis on the role of the RSC within the visual system, recent evidence indicates that visual cues from the primary visual cortex are distinctly represented within the RSC.^232^

Examining the microcircuit network activity using two-photon calcium imaging shows the presence of a stable spatial memory engram in the mouse RSC.^218^ In addition, a series of recent articles have identified visual-landmark cells in the RSC,^147,229,233^ indicating that the RSC can serve as an integration point of visual, motor, and spatial information.^147^ These findings are congruent with known RSC anatomy, given that it is situated at the intersection of areas that encode visual information, motor feedback, higher-order decision making, and hippocampal formation.^117,139,234,235^ The RSC is ideally positioned to integrate these inputs to guide ongoing behavior, and we hypothesize that future studies will further implicate the RSC in other types of behavioral paradigms that require the integration of multi-modal sensory information to guide behavior. This is extremely crucial in the TBI field given that there is evidence showing that TBI patients cannot utilize visual cues to guide behavior such as identifying a hidden platform in a virtual MWM.^236^ Largely, disruptions to the spatial representation of landmarks and visual cues are noticed in TBI patients^236^; however, more work is required to determine the contribution of the RSC to this observation.

## Pre-Frontal Cortex and Non-Hippocampal-Dependent Behavior

As previously described, the RSC has extensive connectivity patterns beyond the hippocampus, and therefore it is not surprising that RSC functions expand beyond those being hippocampal dependent. As evidence of this, RSC dysfunction has also been represented in various non-hippocampal models; for example, in pediatric models of autism, the RSC displays hyperconnectivity with multiple brain areas such as the posterior cingulate cortex,^237^ insular cortex,^238^ and dorsolateral PFC.^238^ Interestingly, these same connections of the RSC have been shown to exhibit hypoconnectivity in conditions of neurotrauma.^239^ Deficits in executive functions and cognition are among the sequela of TBI,^74,240^ and this is, in part, attributable to deficits in the PFC. For example, during the chronic phase of TBI, there is significant evidence showing anatomical alterations and reduced synaptic and axonal integrity in this frontal area.^241,242^ The exact mechanisms of how these alterations contribute to behavioral and cognitive deficits are still yet to be determined. Given the direct anatomical connections between the RSC and PFC, we argue that studying the RSC post-TBI will provide much needed insight into these mechanisms.

Rodent lesioning studies have already shown the close functional association between the RSC and PFC: RSC lesions disrupt the rodent analogue of the Stroop task,^186^ a behavioral task that is sensitive to medial frontal cortex lesions.^243^ In addition, RSC lesions disrupt recency judgments,^227^ another ability closely associated with medial frontal cortex function in rats,^244–246^ as well as cross-modal recognition memory,^226,247^ cross-modal object recognition,^226^ and intradimensional shifts in an attentional set-shifting task,^248^ tasks with a strong frontal function. However, other studies have shown that some frontal tasks do not rely on the RSC. For example, RSC lesions did not disrupt the acquisition part of an intradimensional learning set, affect the ability to switch dimensions,^187^ nor impact cost-benefit discrimination.^187^ Interestingly, some studies have shown an opposite role of the RSC to the one associated with pre-limbic inactivation,^249^ or to not match the one observed after pre-limbic cortex lesions.^250^

These studies highlight the specificity of RSC←>frontal cortex functional interactions and show that RSC lesions spare learning tasks in which there is no mismatch between internal and external representations used to guide behavioral choices. Conversely, RSC lesion deficits emerge on non-spatial tasks when animals have to rely on previously acquired representations to solve the current problem or switch between different representations of the same event.^186,187,205,226,228^

Last, numerous studies have found that the RSC is important for processing pain information; Persons suffering from chronic pain display increased functional connectivity between the RSC and PFC,^251^ a finding that has been recapitulated in rodent models of chronic pain.^252^ Therefore, and, not surprisingly, the RSC, as a major node in the DMN, undergoes functional connectivity alterations in chronic pain models.^253^ RSC functional connectivity with the PFC and subiculum have also been shown to be increased in chronic pain.^252^ These studies set the stage for the RSC as a brain region also involved in non-hippocampal functions. In addition, chronic pain can manifest also subsequent to TBI conditions, particularly in military populations,^254^ potentially offering more context by which further study of the RSC post-TBI is necessary.

Finally, it should be acknowledged that polynodal connectivity of the RSC does produce a measure of difficulty in ascribing where cognitive deficits are centered within the RSC/hippocampal/pre-frontal network. Conceivably, functional deficits at the regional level may occur even in the face of normal levels of inter-regional connectivity because of network compensation as reorganization occurs. Alternatively, deficits in brain nodal communication may occur commensurate with structural white matter injury uncoupled from significant regional alterations, at least acutely. The bottom line is that RSC dynamics should be studied in concert with other brain regions initially and, possibly, using chemogenetics in order to fully understand how the RSC can be used to report back on cognitive deficits after brain injury.

## Conclusion

In recent years, the RSC has begun to attract more attention as a region of major interest in neuroscience. Here, we argue that studying this brain area will be valuable in the field of TBI especially as a proxy or remote reporting conduit for hippocampal and even pre-frontal-related cognitive deficits. Anatomical, functional, and lesion-based evidence indicate a prominent role of the RSC in sensory-cognitive functions, including, most notably, spatial navigation, long-term memory, and sensory integration and experience-dependent plasticity. The central hub connectivity of this region undoubtedly qualifies it as a major region of interest to the TBI field because of the vast number of brain functions that it normally subserves, and that are part of the TBI sequelae that manifest as brain dysfunction.

We suggest that studying RSC functional alterations during functionally burdensome cognitive tasks could provide an important new approach with which to monitor cognitive deficits longitudinally over time after injury, as well as providing an alternative readout for determining the utility of neuromodulatory interventions to improve outcome after TBI. This approach enables the capture of a combination of simultaneous functional-cognitive and behavioral readouts from the same animal and has clear advantages over studying them separately and/or using invasive technology and, possibly, under sedation.

However, because the RSC is highly involved as a sensory-cognitive integrator, it is likely that alteration to the RSC network will have functionally diffuse impacts within the brain. We recommend that the study of cognitive dysfunction through the lens of the RSC be a supplement to the study of regions more traditionally associated with cognition. The superficial location of the RSC lends itself perfectly for use of minimally invasive modalities such as two-photon microscopy and surface-based EEG. In this way, the RSC can be studied as an area of interest without excessive alteration of whole-brain dynamics. Given that TBI and other relevant conditions impact the whole brain, these can be extremely informative tools when used with minimal disruption of the brain. Minimally invasive forms of study will not only make the study of TBI in rodents more accurate, but could also potentially improve the translatability of laboratory findings. The RSC's role as an important node in the DMN, which is marked by altered connectivity after TBI, provides an additional context to drive further study of the RSC in TBI. This would suggest that future studies should consider the RSC as a well-connected, polymodal hub that provides a minimally invasive conduit for studying a variety of cognitive-related deficits after TBI.

## Abbreviations Used

Aβ: amyloid-beta
AD: Alzheimer's disease
DMN: default-mode network
EEG: electroencephalography
fMRI: functional magnetic resonance imaging
MRI: magnetic resonance imaging
MTL: medial temporal lobe
MWM: Morris water maze
PFC: pre-frontal cortex
RSC: retrosplenial cortex
RSCagl: agranular RSC
RSCdys: dysgranular RSC
RSCg: granular RSCg
RSCv: ventral RSC
TBI: traumatic brain injury
